# Multi-Label Attribute Selection of Arrhythmia for Electrocardiogram Signals with Fusion Learning

**DOI:** 10.3390/bioengineering9070268

**Published:** 2022-06-22

**Authors:** Jie Yang, Jinfeng Li, Kun Lan, Anruo Wei, Han Wang, Shigao Huang, Simon Fong

**Affiliations:** 1 Department of Computer and Information Science, University of Macau, Taipa 999078, China; jie.yang@connect.um.edu.mo; 2 Chongqing Industry & Trade Polytechnic, Chongqing 408000, China; steviso@hotmail.com (J.L.); warfantasy@yeah.net (A.W.); 3 College of Mechanical Engineering, Quzhou University, Quzhou 324000, China; 36116@qzc.edu.cn; 4 Faculty of Medicine, The Chinese University of Hong Kong, Hong Kong 999077, China; wanghan@ziat.ac.cn; 5 School of Data Science, City University of Macau, Macau 999078, China; 6 Zhuhai Institute of Advanced Technology (ZIAT), Chinese Academy of Sciences, Zhuhai 519000, China; 7 Department of Radiation Oncology, The First Affiliated Hospital, Air Force Medical University, Xi’an 710032, China

**Keywords:** multi-label attribute selection, arrhythmia recognition, electrocardiogram signals, fusion learning

## Abstract

There are three primary challenges in the automatic diagnosis of arrhythmias by electrocardiogram (ECG): the significant variation among individual patients, the multiple pathologies in the ECG signal and the high cost in annotating clinical ECG with the corresponding labels. Traditional ECG processing approaches rely heavily on prior knowledge, such as those from feature extraction and waveform analysis. The preprocessing for prior knowledge incurs computational overhead. Furthermore, standard deep learning methods do not fully consider the dynamic temporal, spatial and multi-labeling characteristics of ECG data. In clinical ECG waveforms, it is common to see multi-labeling in which a patient is labeled with multiple classes of arrhythmias. However, multiclass approaches in current research mainly solve the multi-label machine learning problem, ignoring the correlation between diseases, resulting in information loss. In this paper, an arrhythmia detection and classification scheme called multi-label fusion deep learning is proposed. The objective is to build a unified system with automatic feature learning which supports effective multi-label classification. First, a multi-label ECG-based feature selection method is combined with a matrix decomposition and sparse learning theory. The optimal feature subset is selected as a preprocessing algorithm for ECG data. A multi-label classifier is then constructed by fusing CNN and RNN networks to fully exploit the interactions and features of the time and space dimensions. The experimental result demonstrates that the proposed method can achieve a state-of-the-art performance compared to other algorithms in multi-label database experiments.

## 1. Introduction

Cardiovascular disease has become the “number one killer” that seriously threatens people’s health. According to statistics released by the World Health Organization (WHO) in 2018, cardiovascular diseases claim 17.7 million lives each year, accounting for 31% of all global deaths [1]. At present, electrocardiogram (ECG-Electrocardiogram) has become an important technology widely used in the inspection and diagnosis of cardiovascular diseases worldwide. The electrocardiogram is a diagnostic technology using electrodes to capture the electrophysiological activity of the skin through the thoracic cavity in time. The electrocardiogram is a technology that records the temporal performance of the heart’s activity over a period of time. The electrocardiogram can reliably reflect the comprehensive state of a beating heart. It is suitable for many biomedical applications, such as heart rate measurement, diagnosis of cardiac abnormalities and even emotional biometrics.

**Figure 1:**
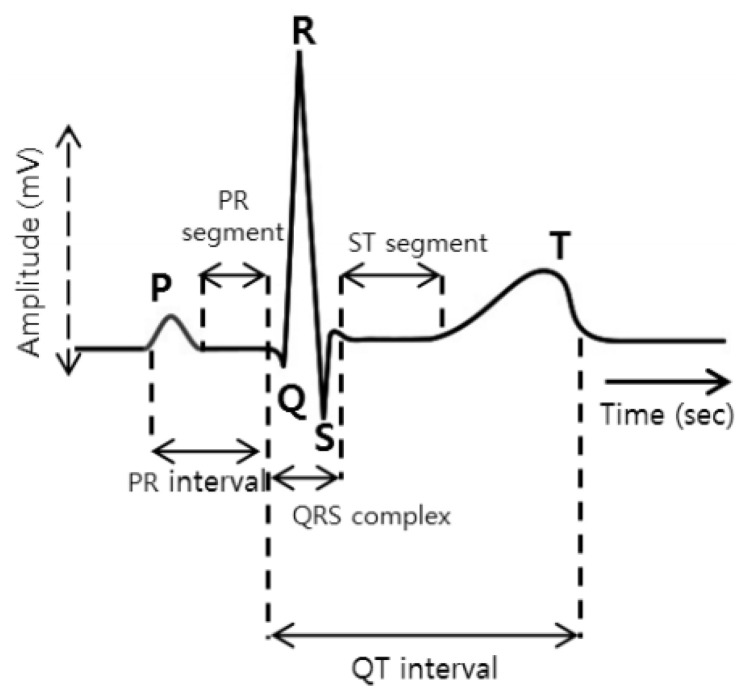
ECG signal wave.

A typical ECG signal is shown in Figure 1. Analysis and manual diagnostics of cardiovascular diseases over a large number of ECG records are known to be very difficult and meticulous. It requires professional knowledge and sophisticated clinical experience. In addition, the diagnosis result may be affected by subjective factors. In order to solve these problems, an automatic ECG classification method is proposed to improve the efficiency and accuracy of diagnosis, and some pioneering work has been done [2]. Current research focuses on single-label classification—support vector machine (SVM), K nearest neighbor (kNN), decision tree and random forest (RF), etc. [3]. The classifier is applied to ECG signal classification [4]. But it is observed that the classification performance is not only determined by the choice of classification algorithm; it is largely dependent on the quality of the ECG data.

ECG signals usually contain multiple cardiovascular diseases at the same time. Therefore, the limitation of one class per label of classical learning (single label) cannot be satisfied. The study of multi-label ECG signal classification is more important than the study of single-label ECG signal classification [5,6].

Traditional electrocardiogram recognition technology includes four parts: electrical signal acquisition, signal preprocessing, feature extraction and electrical signal classification and recognition. At present, the most commonly used electrical signal acquisition method in clinical practice is the twelve-lead method, which includes 6 limb leads and 6 front chest leads, which can record the changes of electrical signals more accurately. The preprocessing of the ECG signal is the prerequisite and basis of the whole ECG recognition. Since the ECG is collected with plenty of noise over a weak electrical signal, it will adversely interfere with the final classification result.

Electrocardiogram preprocessing can filter out interference noises and eliminate baseline drift of electrical signals, such as skin surface noise, respiratory interference, and myoelectric noise. The ECG feature extraction is an intermediate step of the ECG recognition technology. The efficacy of the extraction directly affects the final classification result. When the ECG signal features are extracted effectively, the performance of the classifier will also be significantly improved. Dealing with the high dimensionality of ECG features is one important issue. Mapping ECG signals to multivariate feature space often results in high-dimensional data. In addition, the original ECG features obtained by different ECG feature extraction methods may be redundant or irrelevant for arrhythmia classification tasks. Redundant features may lead to high computational complexity and high-dimensionality catastrophe. Irrelevant features will confuse classification algorithms and reduce learning performance. Therefore, appropriate feature selection is necessary before classifying arrhythmias.

The classification and recognition of the electrocardiogram is the last step of the entire process. It is used as the basis for determining whether the heart rhythm is normal. Classification and recognition are divided into two types: multi-label and single-label. Most research experiments so far are based on single-label classification. Single-label classification is to tell whether a certain disease is present in a patient from a segment of the electrocardiogram. In reality, multiple diseases often co-exist at the same time. Single-label classification cannot precisely reflect the patient’s disease situation. Since the entire process of ECG recognition and classification presents the characteristics of high coupling, dynamics and uncertain labels, so far, few studies have been conducted to deal with multi-label problems throughout the entire process of ECG diagnostic. Therefore, the automatic recognition and classification method of ECG is still a research direction that requires continuous improvement.

The contribution of this research consists of the following parts:

- Firstly, we designed an integrative framework consisting of multi-label feature selection and classification for ECG signals to handle the multi-label and high-dimensionality problems of ECG characteristics simultaneously.
- Secondly, we further developed an effective multi-label arrhythmia classification model for ECG signals. An ECG classification neural network based on feature extraction and time series data processing abilities was constructed.
- Thirdly, by mining the best subset of features among numerous attributes, specific features that can adequately represent the disease association were extracted. The performance of the proposed method was verified to be improved by going through a performance comparison with other multi-label feature selection and classification algorithms.

## 2. Related Work

With the advances of computer hardware and deep learning, the automatic recognition and classification technology of ECG has been widely studied by scholars worldwide. Benefiting from the continuous improvement and establishment of ECG public data sets, more and more ECG recognition and classification methods have been proposed. According to the standards set by the American Association for the Advancement of Medical Instruments (AAMI), heartbeats can be divided into five categories: non-ectopic (N), supraventricular ectopic (SVEB), ventricular ectopic (VEB), fusion heartbeat (F) and unknown heartbeat (Q), in which different heartbeats show significantly different waveforms.

The feature extraction of the ECG signal refers to the extraction of the feature value in the multi-dimensional space of the signal before the recognition and classification of the ECG. Effective feature extraction can lay a good foundation for recognition and classification. If the extracted features are accurate and easy to recognize, the performance of the classifier will be significantly improved, and vice versa.

The feature selection for ECG signals aims to select relevant and indispensable features from the original set of ECG features to form an optimal feature subset while ensuring classification accuracy. It should not only be able to represent the original model to some extent, but also minimize the loss of information. Kamath et al. [7] proposed an energy operator-based feature extraction method with 95% classification accuracy for 67,960 heartbeats. Shen et al. [8] proposed an adaptive feature selection algorithm using wavelet coefficients, which could improve the accuracy of heartbeat classification from 80.32% to 98.92%. However, the dimensionality of the feature space after dimensionality reduction is still greater than 50. Martis et al. [9] compared the effectiveness of three feature selection methods, namely, principal component analysis, independent component analysis and linear discriminant, and analytically validated the performance of probabilistic neural networks for classification on the five beat classifications of arrhythmias (non-ectopic beats, supra-ventricular ectopic beats, ventricular ectopic beats, fusion beats and unclassifiable and paced beats) recommended by the Association for the Advancement of Medical Instrumentation (AAMI). Many early ECG recognition approaches are based on five types of beat classes of arrhythmia. However, due to the limitation of computing power and data volume, the early identification methods cannot reach satisfactory performance. For example, Lin et al. proposed the usage of a weighted linear method for ECG classification on the standard R-R interval and achieved an overall classification performance of 93% [10]. Huang et al. proposed the use of support vector machine (SVM) for recognition and achieved a true positive rate of 90%. The above research method has achieved certain ECG identification and classification goals and improved the ECG computer-assisted diagnosis and treatment technology within a certain range. However, the types of heart diseases are complex and multiple conditions always co-exist. These five coarse particle classifications did not meet the practical application criteria.

Although traditional arrhythmia classification methods use various classifiers to achieve certain results in some test datasets [11,12], they have a disadvantage in processing capabilities or are less researched on complex types, such as time series, multi-label and multi-instance data, etc. Traditional classification methods require pre-knowledge requirements for data waveforms and a large number of operations such as data preprocessing [13] and feature extraction, which hinder large-scale learning and training, and are not conducive to actual clinical applications [14]. Moreover, the above-mentioned traditional methods do not deal with the multi-label problem and do not satisfy actual clinical needs [10].

With the rapid development of computer storage and computing capabilities, the performance of deep learning and neural networks has been considerably improved. Deep neural networks have demonstrated strong detection capabilities in cancer [15], brain diseases [16], Alzheimer’s [17] and other diseases. Due to the “black box” nature of the neural network, it has the advantages of not being necessary to understand the details of the data, high tolerance to data noise and the ability to directly extract the underlying characteristics of the data. Therefore, much complex cardio rhythm detection and classification tasks are naturally solved by using deep neural networks.

Hannun et al. proposed a 34-layers Convolutional Neural Network (CNN) for abnormal heart rhythm detection and obtained cardiologist-level recognition accuracy [18]. Fan et al. fused multi-scale deep CNN to screen atrial fibrillation from single-lead ECG data [19]. Kiranyaz et al. proposed an adaptive CNN model for the detection of patient ventricular ectopic beats and supraventricular ectopics. This model requires only a small amount of data to achieve high accuracy [20]. Acharya et al. used 11-layer CNN to detect myocardial infarction and obtained the best detection performance [21]. Özal proposed a bi-directional long- and short-term memory neural-network (Bi-LSTM)-based model for ECG signal classification. The model is set up with a wavelet sequence layer, which significantly improves the recognition accuracy, achieving 99.39% accuracy in the classification of five heart rhythm abnormalities [22].

In the above-mentioned research work, the timing characteristics of the ECG are usually ignored. Most of the ECG data is split into data blocks for research and learning, and the timing characteristics of the ECG are not considered, and they lack certain clinical practical significance. In order to address the above-mentioned problems, in other studies, the ECG signal is regarded as a time series signal as a consideration and processed by deep neural networks.

Li et al. proposed a model based on deep neural networks and hidden Markov chains to detect intermittent sleep apnea symptoms in ECG signals [23]. Chauhan et al. proposed to use deep LSTM to detect arrhythmia. This model does not need to preprocess the ECG signal to directly generate better detection results [24]. Saadatnejad et al. proposed the use of LSTM to continuously monitor heart rhythm changes through personal wearable devices [25]. Wang et al. proposed a global update heartbeat classification system based on recurrent neural network (RNN) and active learning (Active Learning). The system uses RNN to learn potential features in heart telecommunication signals and uses active learning to update the system to achieve the purpose of recognition and classification [26]. Because RNN has the “memory” advantage in processing time series signals, RNN is the priority choice for processing time series signals. However, the lack of convolution structure will make such models lack the ability to integrate local information and global information and will also make the model lose the ability to build deep layers—network structure ability, leading to partial loss of feature extraction ability. At the same time, a different multi-label classifier has its limitation, although in clinical practice it is common for ECG signals to have multiple diseases [27].

In summary, although ECG research has undergone long-term and extensive research, and has continued to break through with the development of computers, there are still many problems that need to be solved and improved urgently. Traditional ECG processing methods require a strong theoretical basis. They require a large amount of pre-learning knowledge for the data, and each electrocardiogram recognition step needs to be completed independently. The classification result depends on the previous feature extraction and waveform analysis, which is difficult to operate in actual clinical practice.

The neural network method reduces the data preprocessing and feature extraction steps as well as the pre-knowledge requirements for the data, and partially improves the accuracy [28]. However, most of the current algorithms are still not sufficiently developed. Simple CNN fails to consider the characteristics of ECG in the time dimension. Simple RNN lacks the feature extraction ability at the convolutional layer. Although most neural networks can reduce the preprocessing, the preprocessing still significantly improves the final ECG recognition and classification capabilities [29,30]. The mixture of multiple noises will affect the recognition and the feature is difficult to extract [31].

## 3. Methods

### 3.1. Data and Problem Description

**Figure 2:**
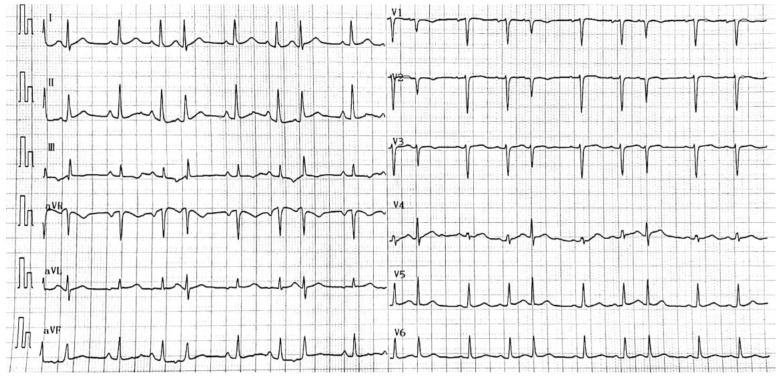
Multi-label ECG signals with Sinus Tachycardia, Right Atrial Abnormality and Atrial Premature Beat Trigeminy.

Table 1 lists the basic information about the dataset. To demonstrate the multi-disease, multi-label nature of the ECG signals implied, Figure 2 shows a 12-conductor ECG’s signals, as illustrated, with the multi-label diseases. This is often the case in the arrhythmia clinical environment.

#### 3.1.1. Dataset and Extraction of Attributes

The organizers of the China Physiological Signalling Challenge (CPSC) [32] collected and integrated clinical data from up to 11 hospitals, which are made openly accessible. The data contains two main parts, the CPSC training set and the CPSC test set. The ECG signal is a 12-lead mode with a sampling frequency of 500 Hz and time duration of 6 to 60 s. The CPSC2018 training set is used as the object of this study because the data set has a multi-label feature and contains a total of 9 kinds of ECG signals, with most of the records having 2 labels and a few even featuring 3 labels. In the proposed model, six ECG signals are focused on; they are the one normal signal and the other five abnormal signals, such as atrial fibrillation (AF), premature ventricular contraction (PVC), premature atrial contraction (PAC), left bundle branch block (LBBB) and right bundle branch block (RBBB). A total of 5078 ECG signal records in the CPSC2018 training set containing five abnormalities (arrhythmias) and one normal ECG record are used in the study. The number of records for each label and the prevalence of each abnormality in the data sample is shown in Table 1. The ratios in the training and test sets are identical, with a random training-test ratio at 7:3. It is noteworthy that the sum of all ratios is greater than 100% due to the presence of the multi-label phenomenon.

The ECG signal shown in Figure 1 should be extracted by first locating the positions of the QRS wave, P wave and T wave of each ECG signal when extracting their characteristics. The algorithm proposed by Datta et al. [33] is used to detect the positions of five waves, which are Q, S, R, T and P waves. A total of 118 features are extracted, based on the different positional characteristics of the Q, S, R, T and P waves. These features are divided into four main types, as shown in Table 2.

#### 3.1.2. Problem Description

According to the above feature extraction method, 118 ECG attribute features $ℱ=\left\{f_{1},\ldots ,f_{27},f_{28},\ldots ,f_{62},f_{63}\ldots ,f_{93},f_{94},\ldots ,f_{k}\right\}$ are extracted. All sample sets formed the attribute feature set $A=\left\{a_{1},a_{2},\ldots ,a_{n}\right\}$ and label set $L=\left\{l_{1},l_{2},\ldots ,l_{m}\right\}$, where k is the number of extracted features, n is the number of samples and m is the number of labels. In order to define the space matrix, we transform the attribute features matrix $ℱ=\left\{f\_td_{i},f\_fd_{j},f\_mg_{k},f\_nl_{l}\right\}$, where f_td denotes the time domain features, f_fd denotes the frequency domain features, f_mg denotes the morphological features and f_nl denotes the nonlinear features, and i∈[1, 27], j∈[28, 62], k∈[63, 93] and l∈[94, 117]. In the ECG signal, we use sliding windows to extract local samples of the ECG signal. The spatial track of each ECG signal is segmented by the windows. Set timestamp $T=\left[t_{1},t_{2},\ldots ,t_{m}\right]$, where $t_{1}$ is the start timestamp and the $t_{m}$ is the end timestamp. Therefore, ${ℱ}_{t1}$ can represent the sample feature matrix representation of this ECG signal under the first window. So each ECG signal can be represented by a two-dimensional matrix:

(1) $${ℱ}_{T1}=\left(\begin{array}{cc} f\_td_{i,t1}\;f\_fd_{j,t1} & f\_mg_{k,t1}\;f\_nl_{l,t1}\\ f\_td_{i,t2}\;f\_fd_{j,t2} & f\_mg_{k,t2}\;f\_nl_{l,t2}\\ \vdots & \vdots \\ f\_td_{i,tm1}\;f\_fd_{j,tm} & f\_mg_{k,tm1}\;f\_nl_{l,tm1} \end{array}\right)$$

Given a series of ECG signals $X=\left[{ℱ}_{T1},{ℱ}_{T2},\ldots ,{ℱ}_{Tm}\right]$, our goal is to construct a discriminable model fusing attribute selection and deep learning to classify the ECG signals with multi-labels. The features associated with the disease labels are first preferred by using the multi-label attribute selection algorithm, and then, these features are fed to the proposed deep learning model as candidate variables. The deep learning model consists of a convolutional neural network (CNN) layer, which can extract feature vectors from ECG data and the set of preferred features, and a gated recursive unit (GRU) layer, which can learn temporal features from m timestamps and perform classification.

### 3.2. Proposed AS-CNN-GRU Model

In this section, we introduce the proposed AS-CNN-GRU model; the attribute-selection-based deep learning fusion structure is developed to handle the multi-label ECG recognition. To better mine the spatio-temporal features of the ECG signal, the two networks, CNN and GRU, are fused. GRU is developed from LSTM networks, both of which alleviate the gradient explosion and gradient vanishing problems during training compared to traditional recurrent neural networks (RNNs). In fact, the use of GRU in this work is motivated by two main aspects: firstly, GRU can remember the state of the previous training process, which is ideal for time series analysis; secondly, GRU has only two gates (i.e., update gate and reset gate) compared to LSTM, so it is more computationally efficient to use GRU and can reach convergence faster [34].

**Figure 3:**
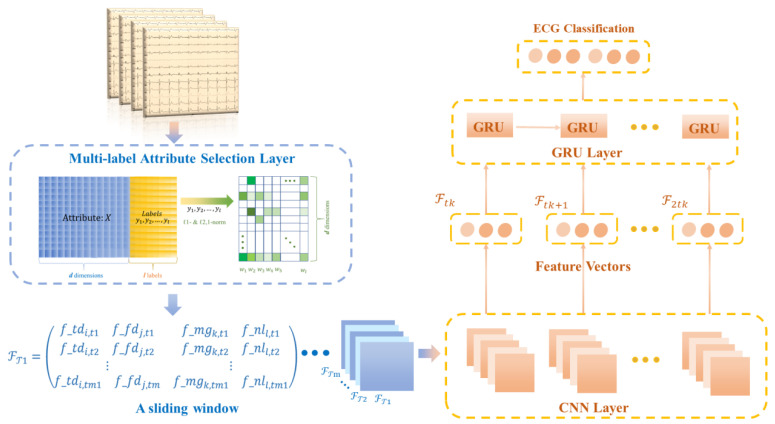
Structure diagram of multi-label attribute selection and classification model for arrhythmia detection.

The structure of the proposed method consists of three parts, as shown in Figure 3. Given the ECG signal and the 118 attribute variables extracted in four categories, the first part is the selection of the disease attributes with the highest correlation through a multidimensional attribute selection layer. The screened attributes and labels are then normalized and transformed into a series of two-dimensional matrices using a sliding-window technique. In the second part, these matrices are used as input to the CNN-GRU layer for the compression and extraction of the implied features between the attribute variables and the disease labels. Finally, multi-label classification of diseases is performed by learning at the spatial and time levels.

#### 3.2.1. Multi-Label Attribute Selection Layer

We use the multi-label attribute selection method [35] to fully explore the shared and specific information between features and labels in ECG data. The goal is to learn the projection matrix association feature space and label space. To extract label-specific and common features for each label, ℓ1-norm and ℓ2,1-norm are used together. The former forces sparsity between all elements and reduces some parameters to zero, which allows for the selection of label-specific features. The latter ensures row sparsity in the matrix and thus avoids losing common feature information.

(2) $$\underset{W}{min}\gamma ∥XW-Y{∥}_{2,1}+\;\gamma_{1}∥W{∥}_{1}+\;\gamma_{2}∥W{∥}_{2,1}\;$$

where *W* denotes the matrix of coefficients obtained in the regression model. $\gamma_{1}$ and $\gamma_{2}$ control the sparsity of the coefficient matrix and the number of common and label-specific features, respectively. Based on the work from Li et al. [36], a common and label-specific feature selection approach for multi-label recognition by learning relevant information about labels and instances is proposed. Cosine similarity and KNN mechanism are applied to evaluate label and instance relevance, respectively. The objective function of this method is summarized as follows

(3) $$\underset{W}{\min}∥XW-Y{∥}_{F}^{2}+\alpha tr\left(FL_{1}F^{T}\right)+\beta Tr\left(F^{T}L_{2}F\right)+\gamma_{1}∥W{∥}_{2,1}+\;\gamma_{2}∥W{∥}_{1}$$

where α, β, $\gamma_{1}$ and $\gamma_{2}$ are constant coefficients. $L_{1}\;$ and $L_{2}$ represent the label Laplacian matrix of *S* and *C*, respectively. *C* is the correlation matrix composed of the instance similarity between each pair of instances evaluated by KNN, and *F* denotes the output matrix.

#### 3.2.2. CNN-GRU Layer

In this layer, the input data of the CNN model is a matrix consisting of the ECG signal and selected feature variables, as shown in Equation (1), where the rows represent the ECG signal values and the features selected from the four types of attributes. Here, ${ℱ}^{\prime }=\left\{f_{1},f_{2},\ldots ,f_{k}\right\}$, (*k* < 118) at a timestamp, and the column denotes timestamp $T=\left\{t_{1},t_{2},\ldots ,t_{m}\right\},t_{m}\in T$. Data normalization is processed using Z-score standardization, as shown in Equation (3).

(4) $$ℐ=\left[\begin{array}{cc} e_{i,t1} & {{ℱ}^{\prime }}_{i,t1}\\ e_{i,t2} & {{ℱ}^{\prime }}_{i,t2}\\ \vdots & \vdots \\ e_{i,tm1} & {{ℱ}^{\prime }}_{i,tm} \end{array}\right]$$

The CNN model mainly contains convolutional and pooling layers. In this study, the CNN model convolutional and pooling layers are used to extract deeper features between the corresponding time length and transformed attributes. The trained feature vectors are then applied to train the GRU layer. Based on the spatial feature information extracted by the CNN, the GRU layer is subsequently used to extract temporal information from these features [37]. The output of the GRU is then fed to the fully connected layer for arrhythmia classification.

**Figure 4:**
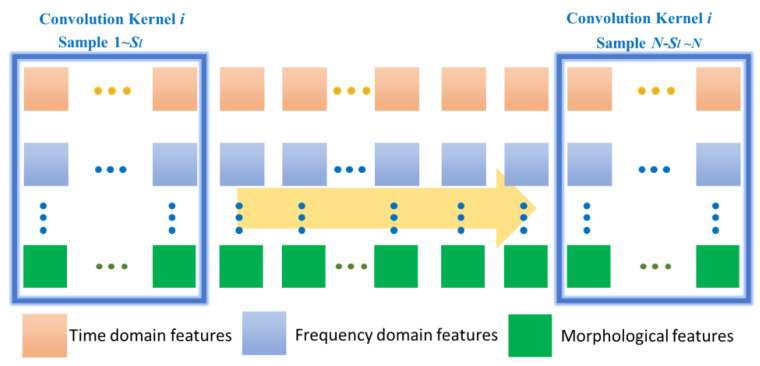
Description of CNN for feature extraction of ECG signal data.

In Figure 4, the *i*th convolutional kernel *K_i_* of size *S_l_* will be slid from sample 1 to N value to extract a feature, and the *l*th feature map *G_k_* can be output as

(5) $$G_{k}=f\left(\sum_{1}^{N}\left(G_{k-1}*k_{i}+b_{i}^{k}\right)\right)$$

where *f* is the activation function, *b* is the *i*th bias of the *k*th feature map, and *N* is the number of convolutional kernels used in the convolutional layer. Assume that the data signal in a single time window can be denoted as *SI_m_*, where *m* is the number of samples in a single time window. In this work, *SI_m_* which can be considered as *G*_0_, the rectified linear unit (ReLU) is usually chosen as the activation function. Since the features extracted by the CNN follow a time series, the temporal information embedded in the ECG signal is preserved and will be used as input to the GRU layer [11]. It is known that stochastic gradient descent (SGD) can help improve the convergence of neural network-based algorithms and make the loss function as small as possible, so the SGD method is utilized in the proposed CNN-GRU-based algorithm.

**Figure 5:**
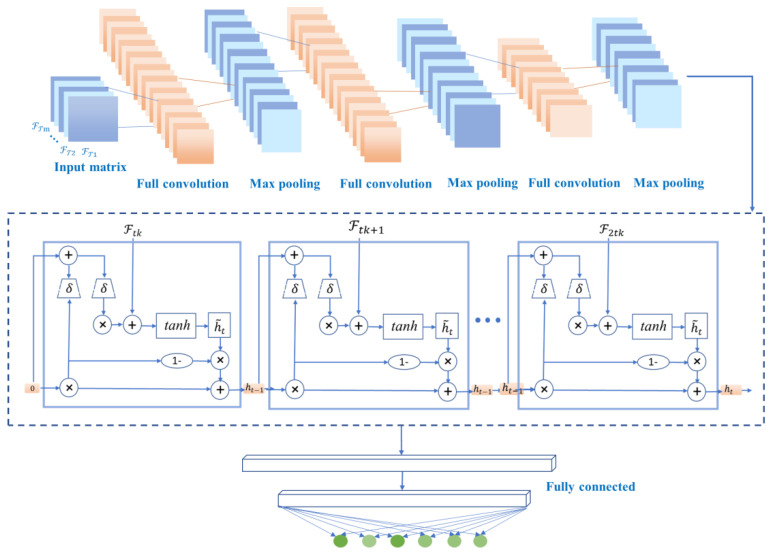
Structural diagram of the proposed CNN-GRU model.

As shown in Figure 5, our proposed ECG detection and classification model is divided into two partial layers. The first part is a multi-label attribute selection layer, and the second part is a CNN-GRU training layer.

In the CNN-GRU training layer, the first input is a series of matrices under timestamps. The convolution layer extracts features from the input matrices, where the convolution acts to maintain the spatial relationships of the variables.

The common methods for pooling layers are maximum pooling and average pooling, which can reduce the number of nodes in the later fully connected layers by reducing the matrix size without changing the depth of the feature map. The model uses maximum pooling. By building multiple convolutional and pooling layers, a complex feature matrix representing the information of each timestamp is extracted for classification, and then the feature matrix is spread into feature vectors to be fed to the fully connected layers. In the proposed model, the CNN model contains two convolutional layers and two pooling layers. And only the feature vectors are used as the input of the next layer. The two convolutional layers and two pooling layers of the CNN model can accurately transform the input data into a feature vector F.

The internal structure of GRU is shown in Figure 5. *Ft* denotes the input sequence of GRU, and *ht* denotes the output sequence, which is the predicted value of GRU. In addition, $r_{t}$, $z_{t}$ and $h_{t}$ are intermediate sequences, which are identified as

(6) $$z_{t}=\sigma \left(w_{z}f_{t}+U_{z}h_{t-1}+b_{z}\right)$$

(7) $$r_{t}=\sigma \left(w_{r}f_{t}+U_{r}h_{t-1}+b_{r}\right)$$

(8) $$h_{t}=z_{t}⊙h_{t-1}+\left(1-z_{t}\right)⊙\tilde{h_{t}}$$

(9) $$\tilde{h_{t}}=\tanh\left[W_{c}f_{t}+U_{c}\left(r_{t}⊙h_{t-1}\right)+b_{c}\right]$$

where *σ* denotes the vector format of sigmoid and tanh denote the hyperbolic tangent functions, respectively. ⊙ denotes the pair-wise operation. $w_{z},\;U_{r},w_{r},\;U_{z},\;W_{c}$ and $U_{c}$ are weight matrices to be trained, and *b_r_*, *b_z_* and *b_c_* are the bias vectors to be trained. In this work, the cross-entropy is selected as the loss function for training. In other words, the optimization model of the proposed CNN-GRU-based algorithm can be represented as

(10) $$minL\left(y,\hat{y}\right)=\frac{1}{N}\sum_{i}^{M}\left[-y_{i}\ln\left(\hat{y_{i}}\right)-\left(1-y_{i}\right)\ln\left(1-\hat{y_{i}}\right)\right]$$

where *y* and $\hat{y_{i}}$ are the actual and predicted labels matrices and $y_{i}$, $\hat{y_{i}}$ are elements of the matrices. *N* is the number of batches in the training process and *M* is the number of feature data sources to be identified. Finally, multi-label classification is performed by minimizing the loss function. In summary, the system model of the proposed CNN-GRU network is shown in Figure 5.

## 4. Results

### 4.1. CNN-GRU Layer

Under the same experimental conditions, the confusion matrix and the following evaluation indicators will be used as performance comparison standards.

Accuracy: The classification accuracy of the model test set can directly reflect the classification performance

ACC = (TP + TN)/(TP + FN + FP + TN)
(11)

Jaccard similarity: It is a measure of distance between the prediction and the ground truth, i.e.,

(12) $$\mathrm{Jaccard}\left(h\right)=\frac{1}{\left|X\right|}\sum_{x\in X}\frac{h\left(x\right)\cap y}{h\left(x\right)\cup y}$$

where h(x) ∩ y is the cardinality of the intersection of vector h(x) and vector y, and h(x) ∪ y is the cardinality of the union of vector h(x) and vector y.

Hamming Loss: It is a label-wise measure that counts the proportion of the labels that are misclassified in all instances, i.e.,

(13) $$\mathrm{Hamming}\;\mathrm{Loss}(h)=\frac{1}{\left|X\right|}\sum_{x\in X}\frac{1}{l}\sum_{j=1}^{l}[(L_{j}\in h\left(x\right))⊗\left(L_{j}\in y\right)]$$

where ⊗ is the logical exclusive—OR, h(x) denotes the classification function, and $L_{j}$ denotes the jth label.

F value: It is the weighted harmonic average of precision rate and recall rate, which can better evaluate the quality of the classification model.

F1 = (2 × Precision × recall)/(precision + recall)
(14)

### 4.2. Results Analysis

**Figure 6:**
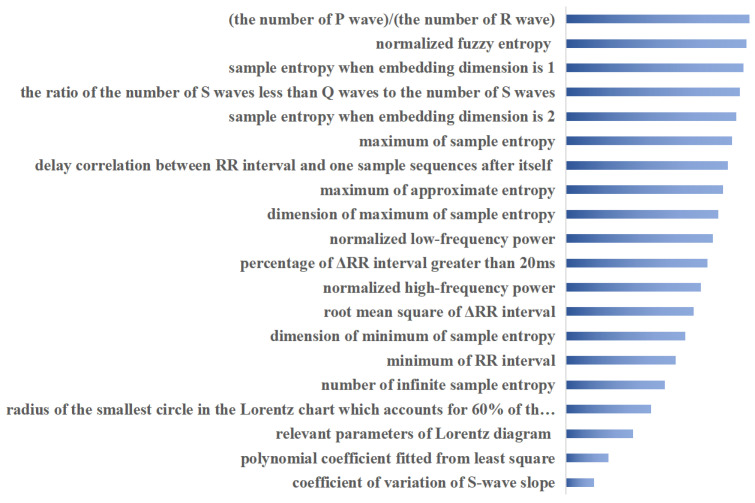
Top 20 important attributes ranked using proposed method.

Based on the ECG features, we trained multi-label ECG signal classifiers respectively, and the specific parameters of the model are shown in Table 3. The proposed attribute selection method selected the most important 20 features for ranking, as shown in Figure 6. The screening of important features combined with clinical diagnostic experience and experimental results give the proposed method more explanatory power. The extracted features are used to train the multi-label ECG classifier.

The training network for the classifier learns the hierarchical features by convolution and pooling operations based on the parameters provided in Table 3. The stochastic gradient descent (SGD) training strategy is used to accelerate the model optimization process, the batch size is chosen as 150 with better performance than other solutions and the learning rate is set to 0.001. In addition, all the network’s parameters are also run through a trial-and-error method at various settings and the best settings are chosen for each network to produce the best performance results. The weights in the model are randomly initialized at the beginning of the training process and progressively updated throughout the process. The classification results are combined to produce the final classification results. For comparison purposes, commonly used classification methods are compared. Purposeful tests are performed on real ECG signal data in order to demonstrate the generalization ability of the proposed method. The extracted features are used to train the multi-label ECG classifier. The fusion model FusionGC and attribute selection-prepared FusionGC (AS+FusionGC) have been utilized separately from the six comparison methods. A total of 60% of the samples are randomly selected for training and the remaining 40% are used for testing, and five-fold cross-validation is used to validate the results. The average classification results based on each multi-label classifier, i.e., BRSVM [4], MLKNN [38], MLHARAM [39], MLSVM [40], Label Powerset [41], Class Chain [42] and LSPC [43], are shown in Table 4. The models marked with an asterisk in Table 4 are methods with added preprocessing and the number of classes is six, as shown in Table 1.

To demonstrate the generalization capability of the proposed method, purposive tests were conducted on real ECG signal data. The proposed attribute selection method selected the most important 20 features for ranking, as shown in Figure 6, and the screening of important features combined with clinical diagnostic experience and experimental results make the proposed method more explainable. To illustrate the efficiency of the proposed fused multi-label classifier, the usual ensemble classifiers are used to analyze the classification performance. As can be seen from Table 4, the multi-label classification results based on the proposed fusion classification method outperformed the respective comparison methods on most of the factors evaluated. Factor accuracy scores, Hamming losses, Jaccard similarity, and F1 scores are all significantly improved. The currently commonly used integrated multi-label classification methods assign the same weight to each classifier and do not take into account the differences between different labels. BP neural network classification models based on ensemble empirical modal decomposition and Fourier transform, classical CNN and LSTM models, and the proposed CNN and GRU fusion model are compared separately. In particular, the effect of the attribute selection method on the proposed classifier model is also compared in Table 4, with more than half of the six metrics compared gaining dominance with the attribute selection preprocessing. This is due to the fusion of the proposed method to learn attribute importance and classification balance. On the one hand, the important attributes are considered more comprehensively, through combination with matrix decomposition and sparse learning theory to fully exploit the shared and specific information between attributes and labels in ECG data. On the other hand, the classification model fully incorporates the learning of dynamic temporal, spatial and multi-labelled features of ECG data, enabling a more comprehensive analysis of the role of the data embedded in the signal.

**Figure 7:**
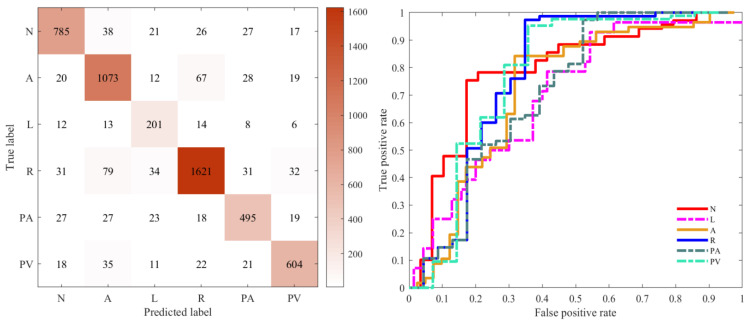
Confusion matrix of the classified performance (**left**). ROC curve of different labels (**right**). The ROC curves for the different disease labels are marked in different colours. (N = Normal, A = AF, L = LBBB, R = RBBB, PA = PAC, PV = PVC).

Figure 7 illustrates the confusion matrix and ROC curves for the multi-label classification performance of the proposed model. Figure 7, left figure, illustrates the confusion matrix for the best accuracy performance of the proposed method for six labels, and the figure illustrates the number of instances of the confusion matrix belonging to a label and not belonging to that label for a given label. For example, AF, the proposed model, produced excellent results on cross-validation, obtaining accurate recognitions for 1073 case labels, representing 88.02%, much higher than the number of false positives and false negatives. The ROC curve shown in Figure 7, right figure, is a graphical representation for showing the trade-off between the true-positive rate and false-positive rate; the classification results of the proposed method are selected for plotting its ROC curve, with the six colors representing the ROC curve for each of the six label classes.

## 5. Discussion

A model called AS+FusionGC is proposed to explore a new method for arrhythmia classification by ECG signals in terms of both data pre-processing and deep feature learning. The model first improves the discrimination of the deep feature learning of the signal by conducting matrix decomposition and sparse learning of the obtained signals. Five out of six performance metrics were achieved by incorporating data pre-processing. Compared to the literature [8,9,10], the proposed approach is closer to the clinical environment, considering the multi-disease and multi-label situation based on the data pre-processing. The proposed model, inspired by the literature [44], uses a network structure idea of spatial + temporal fusion learning, where spatial-information fusion is based on a convolutional neural network and temporal-information fusion is based on a GRU module. These modules with different functions are incorporated into a unified neural network structure to form an end-to-end fusion learning model. The model proposed based on the literature [44] extends the case of multiclass diseases and increases the ability to identify multi-label diseases. However, differences in dataset characteristics lead to an inability to directly compare experimental results. A comparison with the classical spatio-temporal network model CNN+LSTM is performed, and the results show that the proposed model excels in all six performance metrics in Table 4, with four of them being superior. However, the proposed model still has some limitations. Firstly, the amount of data used is relatively limited, and the data is mainly derived from public datasets and needs to be validated with data in more clinical real-world environments. Secondly, the number of multi-label multi-disease cases incorporated into the ECG signal is limited, and more multi-label cases of different disease types need to be evaluated further. Third, the architecture of the model could also take more into account the ability of explainable AI, e.g., the introduction of attention mechanism may improve the diagnostic effectiveness of the model for multi-disease ECG signals. These will be the directions of our future research and efforts.

## 6. Conclusions

In this study, our perspective focuses on the three aspects of spatio-temporal sequence variation, deep attribute selection and multi-label recognition in the ECG recognition process, and the most disease-characterizing attribute features are obtained by fully data mining the multi-disease association information in the extracted features through discriminable attribute selection methods. A hybrid neural network, CNN-GRU, is then established to handle the recognition and classification of ECG, achieving an organic combination of medical and artificial intelligence. New solutions are proposed to improve the multi-label classification and recognition of ECG. The simulation results show that the proposed approach achieves better performance in most cases of multiple metrics testing by refining the attribute features in the ECG signal and fusing deep learning techniques to fully exploit the spatio-temporal features. This method is therefore proven to be useful in the task of ECG multi-label disease classification.

## Figures and Tables

**Table 1:** Selected ECG data characteristics.

Type	Prevalence Rate	Number of Records
Normal	N/A	914
AF	11–15%	1219
PVC	14–16%	711
PAC	5–7%	609
LBBB	5–7%	254
RBBB	5–7%	1828

**Table 2:** Characteristics of the extracted attributes.

Type of Features	No.	Overview	Specific Content
Time domain features	27	A statistical feature is extracted from the RR interval of the ECG signal	the minimum and maximum values of the RR intervals, the median heart rate and the root mean square of the difference between adjacent RR intervals, etc.
Frequency domain features	35	Mainly based on the features of the ECG signal with windows	Calculation of window signal spectrum parameters. Including spectral center, center-of-mass frequency, wavelet transform coefficient, normalized low frequency power and normalized high frequency power, etc.
Morphological features	30	Morphological change features	Calculate the depth of S-wave and Q-wave and R-wave, ST slope, width of QRS, etc. according to the position and amplitude of P-wave, Q-wave, R-wave, S-wave and T-wave.
Nonlinear features	26	Other features	Calculated by nonlinear methods, such as sample entropy, approximate entropy, fuzzy entropy, etc.

**Table 3:** Configurations the proposed CNN-GRU models.

Types	Activation Function	Output Shapes	Kernel Size	No. of Filters	Stride	Trainable Parameters
Input	–	1000 × 1	–	–	–	0
Full convolution	ReLU	1008 × 3	20 × 1	3	1	50
Max-pooling	–	504 × 3	2 × 1	3	2	0
Full convolution	ReLU	520 × 6	10 × 1	6	1	160
Max-pooling	–	260 × 6	2 × 1	6	2	0
Full convolution	ReLU	263 × 6	5 × 1	6	1	160
Max-pooling	–	132 × 6	2 × 1	6	2	0
GRU		20	–	–	–	1280
Fully-connected	ReLU	20	–	–	–	400
Fully-connected	ReLU	10	–	–	–	200
Fully-connected	Softmax	5	–	–	–	55

**Table 4:** Classification results based on different multi-label classification methods.

Methods	Accuracy Score	Hamming Loss	Jaccard Similarity	Precision	Recall	F1
BRSVM	0.411	0.116	0.447	0.519	0.353	0.364
MLKNN	0.560	0.115	0.588	0.72	0.515	0.561
MLHARAM	0.487	0.149	0.625	0.567	0.637	0.552
MLTSVM	0.261	0.143	0.327	0.582	0.369	0.439
Label Powerset	0.718	0.137	0.752	0.854	0.661	0.717
Classifer Chain	0.659	0.068	0.694	0.893	0.584	0.683
LSPC	0.381	0.27	0.376	0.366	0.735	0.486
EEMD + FFT + BP *	0.745	0.072	0.757	0.784	0.736	0.712
CNN + LSTM	0.761	0.07	0.787	0.818	0.745	0.753
FusionGC	0.763	0.06	0.788	0.815	0.748	0.754
AS+FusionGC *	0.774	0.062	0.795	0.839	0.734	0.773

## Data Availability

Not applicable.
